# Can government subsidies and public mechanisms alleviate the physical and mental health vulnerability of China’s urban and rural residents?

**DOI:** 10.1186/s12939-022-01805-2

**Published:** 2023-04-01

**Authors:** Yali Li, Ronald Marquez

**Affiliations:** 1 School of Business, Jiangxi University of Science and Technology, Nanchang, 330013 China; 2 Laboratoire Physico-Chimie des Interfaces Complexes, ESPCI Paris, 10 rue Vauquelin, F-75231 Paris, France

**Keywords:** Government subsidies, Public mechanisms, Physical and mental health poverty vulnerability index, Andersen model, Multivariate logistic regression analysis

## Abstract

**Background:**

Poverty vulnerability has been defined as the likelihood of a family falling into poverty in the upcoming months. Inequality is a major cause of poverty vulnerability in developing countries. There is evidence that establishing effective government subsidies and public service mechanisms significantly reduces health poverty vulnerability. One of the ways to study poverty vulnerability is by using empirical data such as income elasticity of demand to perform the analysis. Income elasticity refers to the extent to which changes in consumers’ income affect changes in demand for commodities or public goods. In this work, we assess health poverty vulnerability in rural and urban China. We provide two levels of evidence on the marginal effects of the design and implementation of government subsidies and public mechanisms in reducing health poverty vulnerability, before and after incorporating the income elasticity of demand for health.

**Methods:**

Multidimensional physical and mental health poverty indexes, according to the Oxford Poverty & Human Development Initiative and the Andersen model, were implemented to measure health poverty vulnerability by using the 2018 China Family Panel Survey database (CFPS) as the data source for empirical analysis. The income elasticity of demand for health care was used as the key mediating variable of impact. Our assessment was conducted by a two-level multidimensional logistic regression using STATA16 software.

**Results:**

The first level regression indicates that the marginal utility of public mechanism (PM) in reducing urban and rural vulnerability as expected poverty on physical and mental health (VEP-PH&MH) was insignificant. On the other hand, government subsidies (GS) policies had a positive suppression effect on VEP-PH&MH to a relatively low degree. The second level regression found that given the diversity of health needs across individual households, i.e., the income elasticity of demand (HE) for health care products, PM and GS policies have a significant effect in reducing VEP-PH&MH in rural and urban areas. Our analysis has verified the significant positive impact of enacting accurate GS and PM policies on effectively reducing VEP-PH&MH in rural as well as urban areas.

**Conclusions:**

This study shows that implementing government subsidies and public mechanisms has a positive marginal effect on reducing VEP-PH&MH. Meanwhile, there are individual variations in health demands, urban-rural disparities, and regional disparities in the effects of GS and PM on inhibiting VEP-PH&MH. Therefore, special consideration needs to be given to the differences in the degree of health needs of individual residents among urban and rural areas and regions with varying economic development. Furthermore, considerations of this approach in the current worldwide scenario are analyzed.

## Background

The World Health Organization (WHO) estimates that approximately 70% of the world’s population still lives in countries where inequality has increased and is at a historical peak [1]. Inequality can exacerbate income, health care, and education disparities between rural and urban areas and country regions, leading to multidimensional poverty in less developed regions [2–4]. Multidimensional poverty reduction is a globally relevant topic that is crucial to achieve the Sustainable Development Goals [5–8]. In the case of China, as a developing country, the goal of fully eliminating absolute poverty was declared as achieved in December 2020 [9, 10]. However, the unbalanced and insufficient development problem in China is still prominent, and relative poverty still exists. In March 2021, China compiled the Outline of the 14th Five-Year Plan for National Economic and Social Development (2021–2025) and 2035 Visionary Goals, in which the equalization of basic public services is mentioned. The latter includes health care, which is continuing to narrow the gap between urban and rural development. Among the main goals, it is pointed out that China has moved to the stage of high-quality development, proposing to consolidate and expand the achievements of poverty eradication. This is allowing to significantly improve the quality of physical and mental health of urban and rural residents, and to reach a new level of the population well-being. Looking into China’s current economic and social status, it has continuously accelerated the development of urbanization, leading to universal coverage of basic health care in urban and rural areas, thus enhancing the health standards of urban-rural residents. Meanwhile, the demand for health services from urban and rural households has strengthened and diversified in the process of urbanization. Consequently, there is a growing contradiction between the increasing pressure on urban-rural households to pay for health care services, the imbalanced supply structure of the health care system, and the inadequate government financial system. The latter makes urban-rural households still suffer from multidimensional poverty in health care, thus, the inequality tends to intensify. Nevertheless, the risk of multidimensional poverty has been a severe threat to Chinese society, and the proportion of potentially poor groups deprived by the vulnerability in public services such as health care and education is increasing significantly [11].

### Governance of poverty

The governance of poverty has gradually evolved from addressing absolute poverty to influencing an individual’s ability to behave in a multidimensional poverty perspective [12]. When people can overcome the minimum threshold of basic security, they may suffer deprivation in other critical areas, hindering personal development and leading to poverty vulnerability [13]. In the actual context of global outbreaks, epidemics, and localized natural disasters, various unexpected risks impact and test the health risk management and response capacity of households, thus weakening the health risk resilience of individuals or families [14–16]. Studies on poverty that adopt an “ex-post evaluation approach” usually only reflect the poverty status of individuals or households in the current period, not allowing to reflect future poverty trends dynamically. In contrast, poverty vulnerability studies focus on the long-term and dynamic nature of poverty, the stability of poverty reduction, and the possibility of returning to poverty, while taking into account the possible future risk shocks faced by individuals and households, and their ability to cope with them [17–19]. Therefore, introducing poverty vulnerability indicators to identify groups at risk of falling into poverty helps the government to formulate effective ex-ante intervention policies for households or regions with higher poverty vulnerability. This is crucial to reduce the probability of poverty occurrence and improve the overall welfare level of society [20].

### Poverty vulnerability studies and risk shocks

Poverty vulnerability studies are essential for policy formulation in developing countries [21]. The World Bank formally introduced the concept of “poverty vulnerability” in 2001, defining it as the likelihood that the future welfare of an individual or household will fall below a socially acceptable level in response to a risk shock [22, 23]. While foreign research on poverty vulnerability began in 2001, Chinese scholars began to study it only after 2009 [24]. The early literature on poverty vulnerability in economics measured the vulnerability of individuals or households based on their exposure to risk shocks [25], examining the extent to which various sources of shocks affect consumption expenditures. Later, researchers looked at poverty vulnerability in terms of expected welfare [26]. Thus, there are three main approaches to measure poverty vulnerability in the currently available literature, both domestically and internationally. The first method is Vulnerability as uninsured Exposure to Risk (VER), which measures using risk exposure facts [27, 28]. The second is Vulnerability as low Expected Utility (VEU), measuring welfare loss in terms of changes in effects [21, 29]. The third is Vulnerability as Expected Poverty (VEP), which measures ex-post the probability of future poverty [30]. The three allow to measure poverty vulnerability by analyzing the likelihood that the studied population will fall below the poverty line in the future [31, 32]. The VEP measure is an ex-ante prediction of poverty vulnerability from an expected poverty perspective, which can be satisfied by cross-sectional data and is widely used. Given the approach of VEP assessment, the vulnerability analysis should consider the response and capacity of households to cope with risks. Therefore, the direction of the poverty vulnerability measure is extended from measuring single dimensions of income and consumption to measuring multiple dimensions concerning the living status and health status of households. This measure evolution allows a more individualized and dynamic nature to the vulnerability assessment [33, 34].

### Anti-poverty vulnerability policies

How anti-poverty vulnerability public policies are implemented, particularly how specific assistance policies are formulated and executed, is crucial to overcome the current poverty challenges. Taking the example of health poverty that an individual or a family may expect to suffer in the future, the government needs to reduce health poverty vulnerability through financial subsidies [35]. The economic and public health inequalities between urban and rural areas and between regions are the main drivers of health vulnerability poverty in developing countries [35]. The economic development of the central region tends to be at the expense of the peripheral regions. At the same time, the local government is prone to a strong bias in providing public health services to the central region in pursuit of competitiveness. Therefore, it has been found effective for the government to reduce health poverty vulnerability through financial subsidies [19]. Simultaneously, it is also necessary for the government to diminish health poverty vulnerability by formulating and implementing public policies. The government has increased the supply of health services to local families or individuals by giving them special accommodations and preferences in health care that can help health poverty vulnerability families or individuals to break out of the poverty vulnerability cycle [36]. However, some researchers have found that health poverty vulnerability may vary between rural and urban areas or between regions [35, 37]. The severity of deprivation from health poverty vulnerability may also vary across regions due to inter-regional development disparities, and the level of need for physical and mental health may also vary. Thus, reducing health poverty vulnerability through non-differentiated government financial subsidies and public policy interventions is not an adequate approach [38, 39].

### Physical and mental health vulnerabilities

Ward [21] argues that the degree of need for physical and mental health is quite different by gender, education level, social relationships, and risk attitudes in each region. Therefore, considering these differences, an accurate public mechanism should be designed for poor and vulnerable populations [40]. Several studies have found that socio-demographic characteristics, social relationship characteristics, and personal habits are also considered essential factors in physical and mental health [40, 41]. In European countries, female gender, older age, lower education level [42], lower social status [43], and unmarried or living alone [44] are important factors associated with physical and mental health. This situation is also more noticeable in developing countries, especially China [40, 45–48]. It is also the case that social relationships with mutual interaction and economic support are beneficial for physical and psychological health [49–52]. Given the worldwide context of climate change, outbreaks, the recent pandemic and regional conflicts, changes in policies regarding containment and closure, closed or semi-closed social relationships may also have impacted physical and mental health status [41]. Therefore, it is of academic meaning and research value to consider the variations in personal and social characteristics that may affect health, when developing and implementing a differentiated public policy. This is performed in combination with attention to the differences in the degree of physical and mental health demands of each household or individual. Thus, incorporating them consistently into a research framework that affects vulnerability to health poverty.

Although theoretical studies on health poverty vulnerability are relatively common nowadays, herein we measure health poverty vulnerability by focusing on China as the research object, compiling and analyzing the 2018 China Household Survey Study (CFPS) database as the data source. Government financial subsidies were analyzed through a dual-layer multidimensional logistic regression model. These are evaluated before and after considering different levels of physical and mental health needs of individual residents between urban and rural areas, by employing the income elasticity of health demand as an essential mediating joint effect variable. The results of this study can be used to analyze the impact of government financial subsidies and public mechanisms on physical and mental health poverty vulnerability. The results of this study have important implications for developing countries in addressing the health poverty problem caused by imbalances, even more with the current unpredictability and instabilities due to climate change, outbreaks, regional conflicts and the remaining COVID-19 challenges [53, 54]. We believe that the results of this study are enlightening for developing countries to address the health poverty caused by imbalances, and consequently, deploy adequate government policies.

## Methods

### Data sources

The study sample was drawn from 2018s China Family Panel Studies (CFPS). The CFPS was implemented by the China Social Science Survey Center (ISSS) of Peking University. It collected information on a range of variables at three levels: individual, household, and community. The individual database includes basic information (age, gender, marriage, family size, etc.), education, work, and income, social relationship, financial subsidy, and health (perception of physical and mental health, evaluated health, access to health insurance, health expenditure, and health service utilization).

**Fig. 1:**
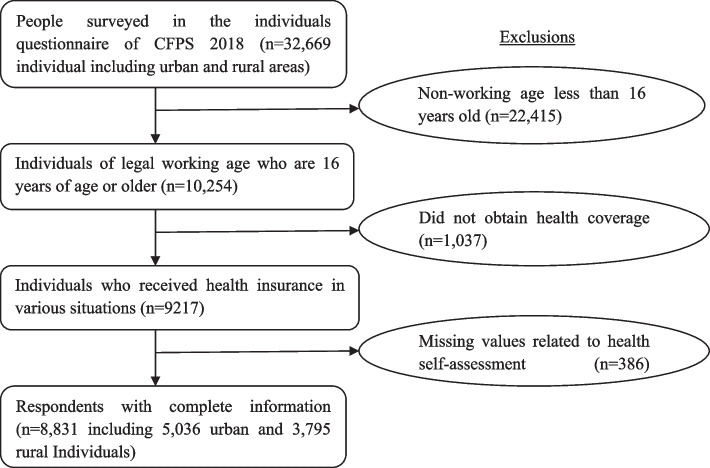
Sample size determination (*n* = 8831) from the 2018 China Family Panel Studies (CFPS) survey

The 2018 CFPS survey adopted a multi-stage probability sampling, with more than 1800 villages in 30 provinces and cities of China as the primary sampling units. It recruited 13,996 households, including 7252 urban and 6744 rural households, with a total sample of 32,669 individuals, comprising 16,191 urban residents and 15,954 rural residents with 524 missing values. Our sample was restricted to urban and rural individuals who benefited from China’s current anti-poverty policies. However, for many reasons, in collecting data on individuals’ total income and work status, a sample of 22,415 age groups not applicable to work was excluded (legal working age in China refers to being 16 years of age or older). Also, the data of 1037 residents without health insurance access and 386 respondents with missing values of health expenditure and perception of physical and mental health were excluded. This resulted in a database of 8831 individuals with 5036 urban and 3795 rural residents. In Fig. 1, a detailed sample size determination is shown. Ethical approval was not required for the research involving secondary use of CFPS de-identified data.

### Specification of the empirical model

A two-layer multiple logistic regression model is used for cross-sectional data analysis and selects physical and mental health vulnerability as the dependent variables based on Andersen’s health care utilization conceptual model [55]. The selection of independent variables for the two-layer regression analysis was performed by taking financial subsidies from local governments and the level of public mechanism for health services as the independent variables for the first layer. Then, it takes the income elasticity of demand for health and the interactive variables related to elasticity as the independent variables for the second layer [56]. The first layer of the multiple logistic regression models for cross-sectional data can be constructed as follows (Eq. 1):

1 $${\displaystyle \begin{array}{c}{y}_n^s=\frac{1}{1+{e}^{-{\vartheta}_n}},s= physical, mental\\ {}{\vartheta}_j={\beta}_0+{\beta}_1{x}_1+{\beta}_2{x}_2+\cdots +{\beta}_n{x}_n+\mu \left(n=1,\cdots, m\right)\end{array}}$$

Where *x*_*n*_ is set as the key impact explanatory variables; *ϑ* denotes the remaining covariates; *β*_*n*_ is the parameter vector corresponding to the explanatory variables; *β*_0_ is the individual item; μ is the residual item estimated by the model; and ${y}_n^s$ is the unobservable variable (latent variable). When ${y}_n^s$ > 0, it is recorded as 1; otherwise, it is recorded as 0.

The second layer of the multiple logistic regression model for panel data can be constructed as follows (Eq. 2):

2 $${\displaystyle \begin{array}{c}{\beta}_{0n}={\gamma}_{00}+{\gamma}_{01}E\_H+{\varepsilon}_1\\ {}{\beta}_1={\gamma}_{10}+{\gamma}_{11}E\_H+{\varepsilon}_1\\ {}\begin{array}{c}\vdots \\ {}{\beta}_n={\gamma}_{n0}\end{array}\end{array}}$$

Where *E* _ *H* is the key variable added in the second layer, i.e., the income elasticity of demand for health care variable, and the detailed measurement process of *E* _ *H* is stated in the independent variable section below. To obtain the interactive variables for the second layer, by merging Eq. (2) into Eq. (1), Eq. (3) is obtained:

3 $${\displaystyle \begin{array}{c}{y}_n^s=\frac{1}{1+{e}^{-{\vartheta}_n}},s= physical, mental\\ {}{\vartheta}_j={\gamma}_{00}+{\gamma}_{01}{E}_H+{\gamma}_{11}{E}_H+{\gamma}_{11}{X}_i\times E\_H+\cdots +{\gamma}_{n0}\ {x}_n+\mu +{\varepsilon}_n\left(n=1,\cdots, m\right)\end{array}}$$

In the second layer logistic model, the structural influence of the second-stage factors on *x*_*n*_ is attained by focusing on the coefficient *γ*_11_, which is expressed as the degree of change in the effect of the key variable *x*_*n*_ on the dependent variable ${y}_n^s$, in the first stage of the logistic model. If the second-stage key variable *W*_*j*_ changes by 1%, then *γ*_11_ is the interaction term connecting the first stage with the second stage. If *ρ*_11_ is significant, it reflects that the key explanatory variables in the second level impact the effect of the first layer *x*_*n*_ on ${y}_n^s$.

For panel data use, there are three main types of multiple selection models, i.e., pooled, random effects, and fixed effects multiple logistic regression models [25]. We selected the fixed effects logistic method for our baseline results based on the Hausman specification test results. All data analyses were performed using STATA 15.0 (StataCorp LP, College Station, Texas).

### Dependent variable

The key-dependent variables are first constructed to perform the analysis. It contains the expected poverty vulnerability index for both physical and mental health care for urban and rural residents in 2018. Basic information built on individual residents (including household head code identification, age, gender, marriage, education level, urban-rural classification, and family size), total annual income information, and total annual expenditure information on health care were collected from the CFPS household economic module and individual module. Then, individual households’ physical and mental health multi-poverty index was estimated by a multidimensional health care poverty identification method [57, 58]. Finally, the vulnerability as expected poverty on physical and mental health (VEP-PH&MH) of individual households in urban and rural areas was estimated by the Vulnerability as Expected Poverty (VEP) method, based on the information of these variables [30]. The basic formula for calculating vulnerability, according to Chaudhuri et al. [30] and Christiaensen et al. [59] is as follows (Eq. 4):

4 $${VUL}_{ht}={P}_r\left({C}_{h,t+1}\le poor\right)$$

where welfare is defined in terms of consumption so that the household vulnerability of h at a time t- *V*_*ht*_- is the probability that the household’s level of consumption at time t + 1 (*C*_*ht* + 1_) will be below the consumption poverty line.

Consumption t + 1 can be expressed as the observable variables (*X*_*h*_) and the functions of error terms containing shock factors (*e*_*h*_). The formula for consumption t + 1 is as follows (Eq. 5):

5 $${C}_{h,t+1}=f\left({X}_h,{\alpha}_h,{e}_h\right)$$

The estimation strategy presented by Chaudhuri et al. [30] and the three-stage feasible generalized least-squares method by Amemiya [60] are used.

The first step in the calculation is to estimate the consumption equation (Eq. 6):

6 $$Ln{C}_{h,t}={\alpha}_h{X}_{h,t}+{e}_h$$

where *C*_*h*, *t*_ represents the consumption of individual h in the t period, *X*_*h*, *t*_ expresses some individual or family characteristic variables. Thus, among the variables included are age, education level, gender, family size, among others. The predictive dependent variable $\hat{C}={C}_{h,t}$ and residual term *σ*_*e*, *h*_ can be obtained by using Eq. (6).

The second step estimates the sum of the expected $\hat{E}$ and variance of ${\sigma}_{e,h}^2={X}_h\beta$ for the logarithm consumption expressed as (Eq. 7 & 8):

7 $$\hat{E}=\left(\ln {C}_h\left|{X}_h\right.\right)={X}_h\hat{\alpha}$$

8 $$\hat{V}\left(\ln {C}_h\left|{X}_h\right.\right)={\sigma}_{e,h}^2={X}_h\hat{\beta}$$

The third step assumes that consumption obeys a normal distribution; then, the vulnerability calculations can be reduced to the following (Eq. 9):

9 $$\hat{{\textrm{VUL}}_{\textrm{h}}}=\hat{{\textrm{P}}_{\textrm{r}}}\left(\ln {C}_h\le \ln \textrm{poor}\right)=\upvarphi \left\{\ln \textrm{poor}-{X}_h\hat{\alpha}/{\left({X}_h\hat{\beta}\right)}^{1/2}\right\}$$

It is worth noting that the calculation of multidimensional physical and mental poverty indices is crucial in determining the estimated VEP-PH&MH index. The present work collects the health care utilization identification designed in the Andersen model [55, 61, 62], which has guided systematic investigations into the factors that lead to the use of health services. Thus, it includes the predisposing, enabling, and need factors, and identifies and estimates the physical and mental health poverty of individual households in urban and rural areas.

The identification method consists of three steps in the A-F method [30]. The critical deprivation vector of indicator “a” is determined at the first step, that is *G*_*a*_ = (*G*_1_, *GZ*_2_, ⋯, *G*_*n*_)^*t*^. It is assumed that ${\varphi}_{ab}^t$ is an identification value for a single vector, for any matrix ${Y}_{\times n}^T$. When ${y}_{ab}^t<{G}_a$, it indicates that the household “m” is recognized in poverty level during the t-period and counted as ${\varphi}_{ab}^t=1$, otherwise, counted as ${\varphi}_{ab}^t=0$. The second step is to determine the indicator weight vector, i.e., *W*_*a*_ = (*w*_1_, *w*_2_, ⋯, *w*_*n*_). The W_a_ is the nth indicator weight and meets the condition of the equation, i.e., $\sum_{a=1}^j{w}_n=1$. Then by constructing the equation of the weighted deprivation matrix K, i.e., ${k}_a^t=\sum_{\textrm{b}=1}^{\textrm{n}}{\textrm{w}}_{\textrm{n}}{\varphi}_{ab}^t$, the individual households “b” was deprived of the score on all the indicators during the t-period. At the third step, the multidimensional poverty threshold vector “ γ ” is set, and when the condition is reached, i.e., ${k}_a^t\ge \upgamma$, the individual household “b” is recognized as a multidimensional poor household during the t-period. The multidimensional dimension of poverty in physical health and mental health selected in this study is obtained according to the context of China’s actual development by referring to the Multidimensional Poverty Index (MPI) system conducted by the Oxford Poverty & Human Development Initiative (OPHI) in the UK [63] and based on the Andersen health care model [55, 61, 62].

Based on the previous studies showing that individual residents in China’s urban and rural areas are deprived of physical and mental health care, and the availability of CFPS database data. Herein, we design physical health poverty (PHP) and mental health poverty (MHP) indicators and weights, according to the predisposing, enabling, and need factors in the Anderson health care model. The PHP identification adopts “a break on weekends” as the predisposing factor and “having access to health insurance” as the enabling factor. If the answer to both indicators above is “no”, the probability of poverty is presumed to be increased, which is expressed as “1”, and if the answer is “yes,” the probability of poverty is assumed to be reduced and is assigned “0”. Then, the PHP identification selects “whether you were unwell in the past two weeks,” “whether you had chronic diseases in the past six months,” “whether you had bronchitis in the past six months,” “whether you had asthma in the past six months,” “whether you were hospitalized in the past year,” as the need factors. The above five indicators mainly measure the individual PHP by illness and hospitalization. If the answer of the indicator is “yes”, it is assigned “1” if the answer is “no,” it is assigned “0”.

The MHP identification selects “satisfactory social relationship” as the predisposing factor and “having access to health insurance” as the enabling factor. If the answer to both indicators is “no,” the poverty probability is presumed to be increased, which is expressed as “1”; and if the answer is “yes,” the probability of poverty is assumed to be reduced and is assigned “0”. And the MHP identification selects eight indicators from psychological survey questions QN406-QN420 in the “Behavior and Mental State” module of the individual questionnaire as the need factors. These eight questions are all scored 1–4, where a score of 1–2 is presumed to be the absence of mental illness, and a score of 3–4 is presumed to be the presence of some degree of mental illness. The latter is based on the principle of consistency in evaluation criteria “0, 1”, thus the score of 1–2 is recorded as “0” and the score of 2–4 is recorded as “1”. According to the above selection of metrics for PHP and MHP, there are seven and ten indicators to measure the identification of PHP and MHP in three dimensions, respectively (see Table 1 and Table 2). It is worth noting that the design of weights for poverty identification is based on the Alkire et al. [63] weight setting method. Three dimensions are assigned equal weights, reflecting a normative judgment of equal importance to capture multidimensional poverty. Finally, the multi-dimensional poverty rate is set according to the official poverty threshold (deprivation in three or more indicators or *k* = 30%). When the proportion of the seven and ten indicators values exceeds 30%, it is considered that the individual household is in multi-dimensional physical health poverty (PHP) and mental health poverty (MHP) state, i.e., *m* = 1) and (*m* = 0), respectively.

**Table 1:** Multidimensional PHP Indicator System for Health Care

Dimensions	Indicators	Threshold of deprivation	Weight
Enabling	health insurance	Access to health insurance (Yes = 0, No = 1)	1/7
Health need	Chronic illness	Whether or not chronic illness has been in the last 6 months (Yes = 1, No = 0)	1/7
	Bronchitis illness	Whether or not bronchitis illness has been in the last 6 months (Yes = 1, No = 0)	1/7
	Asthma illness	Whether or not asthma illness has been in the last 6 months (Yes = 1, No = 0)	1/7
	Hospitalization	Whether or not entering the hospital in the past 12 months (Yes = 1, No = 0)	1/7
	Health status	Whether you were unwell in the past 6 weeks (Yes = 1, No = 0)	1/7
Predisposing	Degree of work strain	Has a break on weekends? (Yes = 0, No = 1)	1/7

*Source*: China Family Panel Studies (CFPS) 2018, based on A-F method and Andersen model [55, 57, 64]

**Table 2:** Multidimensional MHP Indicator System for Health Care

Dimensions	Indicators	Threshold of deprivation	Weight
Enabling	health insurance	Access to health insurance (Yes = 0, No = 1)	1/10
Health need	Degree of depression	1–2 score as a low degree of depression, 3–4 score as a high degree of depression (1–2 score = 0, 3–4 score = 1)	1/10
	Degree of struggle to do anything	1–2 score as a low degree of struggling, 3–4 score as a high degree of struggling (1–2 score = 0, 3–4 score = 1)	1/10
	Degree of poor sleep quality	1–2 score as a low degree of poor sleep, 3–4 score as a high degree of poor sleep (1–2 score = 0, 3–4 score = 1)	1/10
	Degree of unpleasantness	1–2 score as a low degree of unpleasantness n, 3–4 score as a high degree of unpleasantness (1–2 score = 0, 3–4 score = 1)	1/10
	Degree of loneliness	1–2 score as a low degree of loneliness, 3–4 score as a high degree of loneliness (1–2 score = 0, 3–4 score = 1)	1/10
	Degree of sadness	1–2 score as a low degree of sadness, 3–4 score as a high degree of sadness n (1–2 score = 0, 3–4 score = 1)	1/10
	Degree of difficulty	1–2 score as a low degree of difficulty, 3–4 score as a high degree of difficulty n (1–2 score = 0, 3–4 score = 1)	1/10
	Degree of loss of desire to live	1–2 score as a low degree of loss of desire to live, 3–4 score as a high degree of loss of desire to live n (1–2 score = 0, 3–4 score = 1)	1/10
Predisposing	Satisfactory social relationship	A satisfactory social relationship (Yes = 0, No = 1)	1/10

*Source*: China Family Panel Studies (CFPS) 2018 based on A-F method and Andersen model [55, 57, 64]

### Independent variable

In this stage, the key independent variables are built. It is divided into two sets of variables. The first is composed of government subsidies (GS) and public mechanisms (PM). The CFPS family economic survey is used to collect whether the rural household received the GS, and individual households can be matched to receive GS by using the family code in the household panel. Thus, the GS index is assigned as “1” for individual households receiving the GS. At the same time, other situations are marked as “0”. The level of supply of local public health care resources, i.e., the effectiveness of PM can be estimated from the medical evaluation section of the individual questionnaire module. This is performed by using two indicators: (i) respondents’ satisfaction with the conditions of visiting public hospitals in the urban or rural areas (where they are classified), and (ii) the health care level in public hospitals, reflecting the inequality of local PM in urban and rural areas. The PM is measured on a scale of 1–10, with lower scores indicating that respondents perceive public mechanisms in their area to be less equal.

The second part comprehends the determination of the income elasticity of demand for health care (E_H), the interaction variables (IVS) between government subsidies with the elasticity (GS*E_H), and the interaction between public mechanisms with the elasticity (PM*E_H). This is performed to define the income elasticity of demand for health care. The “income elasticity of demand for public goods” used in this study focuses on the income elasticity of demand for health for rural and urban residents, respectively. The latter can be expressed as the proportion of the rise (or fall) in demand for health when household income rises (or falls) by 1%, ceteris paribus. Demand, in this case, represents the demand that residents have the affordability and willingness to achieve, i.e., the demand within each resident’s budget constraint, and can be measured in monetary payments [64]. As a result, the monetary payment for health by urban and rural residents can be used as the demand for health services.

Based on the annual resident health expenditure obtained from the individual economy module in the CFPS database, urban and rural households’ demand for health care can be estimated. The latter is expressed as health, and the annual income of urban and rural individual households is used as the amount of income to consume health care services, which is expressed as I. The income elasticity of demand for health care for urban and rural individual households is formulated as follows (Eq. 10):

10 $${\textrm{E}}_{\textrm{H}}=\frac{\frac{{\Delta \textrm{Q}}_{\textrm{health}}}{{\textrm{Q}}_{\textrm{health}}}}{\frac{\Delta \textrm{I}}{\textrm{I}}}=\frac{\Delta {\textrm{Q}}_{\textrm{health}}}{\Delta \textrm{I}}\times \frac{\textrm{I}}{{\textrm{Q}}_{\textrm{health}}},\left(\textrm{E}\_\textrm{H}\ge 0\right)$$

A dual logit model was constructed to study the relationship between urban and rural individual household income and health care demand. The natural logarithm of urban and rural individual household income was used as the independent variable and the natural logarithm of health care demand as the dependent variable, and the basic regression equation was established to obtain the coefficients of the independent variables. This was performed in order to estimate the income elasticity of demand for health care. The model is as follows (Eq. 11):

11 $$\ln {\textrm{Q}}_{\textrm{health}}=\upalpha +{\upbeta}_{\textrm{h}}{\textrm{In}}_{\textrm{Y}}+\upvarepsilon$$

In Eq. (11), β_h_ represents the income elasticity of demand for health.

### Covariates

Considering the influence of other factors on VEP-PH&MH and minimizing geographical and instability of regression results, this study included personal information identification (PII), social relationship identification (SRI), health-related lifestyle habit identification (LHI), and level of trust in health illness visits (PT) in the analysis framework. The latter included a) individual household registration, gender, age, family size, marital status, and years of education as PII; b) job satisfaction, social status, used as SRI; c) smoking, alcohol habits, and exercise, used as LHI; and d) patients’ trust to physicians, considered as PT. The basic descriptions and statistical results of each variable are shown in Table 3.

**Table 3:** Variable description and statistics

Identification	Variables	Description	Mean	SD^a^
VEP-PH&MH	VEP on physical health and mental health	The estimation of VEP: With physical and mental vulnerability, VEP = 1. Without physical and mental vulnerability, VEP = 0	0.953	0.211
GS	Government subsidies	Government subsidies: Receiving government subsidy, GS = 1. Non-receiving government subsidy, GS = 0	0.335	0.472
PM	Public mechanism level	Public mechanism level: value ranges:1-10, meaning from low to high	0.6417	0.480
E_H	E_H	Income elasticity of demand for health: The higher the HE, the greater the effect of the degree of the population’s demand for health on changes in their income and vice versa	0.417	0.343
IVS	GS*E_H	The interaction variable between government subsidies with elasticity	0.150	0.289
	PM*E_H	The interaction variable between public mechanisms with elasticity	0.385	0.347
PII	Gender	Gender: male = 1, female = 0	0.602	0.490
	Age	Age, actual age, value ranges: 16–96	39.126	12.103
	Family size	Family size, value ranges:1–17	3.908	1.993
	Marriage	Marital status, with spouse = 1, without spouse = 0	0.803	0.398
	Education	Years of education, from illiterate to doctorate, value range: 0–22	10.503	4.116
SRI	Job satisfaction	Job satisfaction level, value range:1–5	3.638	0.862
	Social status	Social status, range: 1–5, meaning from low to high	2.922	0.984
LHI	Smoke	Smoking: Smoking in the past month? Yes = 1, No = 0	0.360	0.480
	Drink	Drinking: 3 times per week in the past month? Yes = 1, No = 0	0.174	0.379
	Exercise	Exercise, the frequency of physical activity in last week, value range: 0–50	3.281	7.460
PT	Patient trust	Patients’ level of trust in the proficiency of doctors, value range: 0–10	6.539	2.289
IIR	Urban	Individual Household registration: urban = 1, rural = 0	0.650	0.477

^a^*SD* standard deviation

## Results

### First-layer multivariate logistic model regression analysis: marginal effects of government subsidies and public mechanisms on vulnerability as expected poverty on physical and mental health (VEP-PH&MH) of individual residents in rural and urban China

This section is divided into two aspects (i) the marginal effects of government subsidies and public mechanisms, and (ii) the marginal effects of covariates and area, both related to VEP-PH&MH.

### The marginal effects of government subsidies and public mechanisms on VEP-PH&MH

Table 4 presents the logistic regression analysis of the cross-sectional data from the 2018 CFPS survey. We used cross-sectional data from the 2018 CFPS wave and analyzed the impact of the baseline regression. This includes the marginal utility obtained from regressions of the main explanatory variables (GS and PM), and the marginal utility obtained from regressions controlling other key variables (items 3–14 of the table). In addition, based on the inequality of public health service resources and economic levels in rural and urban China, the impact of urban versus rural areas (IIA) is also analyzed.

**Table 4:** Variable description and statistics

Item	Identification VEP-PH&MH	Variables	dy/dx (%)	z
1	GS	Government subsidies	-0.0451^*^	-9.29
2	PM	Public mechanism level	-0.0015	-0.32
3	PII	Gender	0.0095	1.70
4		Age	-0.0010^***^	-4.12
5		Family size	0.0222^***^	11.96
6		Marriage	-0.0110^*^	-1.72
7		Education	-0.0007	-1.15
8	SRI	Job satisfaction	0.0048	0.18
9		Social status	-0.0004^*^	-1.65
10	LHI	Smoke	0.0028	0.05
11		Drink	0.0173^**^	2.50
12		Exercise	-0.0003	-1.17
13	PT	Patient trust	-0.0007	-0.67
14	UDV	Urban	-0.0167^***^	-3.23
15	Number of samples		8831	
16	Province		Yes	
17	Prob >F		0.000	

*Source*: China Family Panel Studies (CFPS) 2018 [64]

*Note*: ^*^、^**^、^***^ represent statistic indicators significantly at 10, 5, and 1%, respectively

The study revealed that Government subsidies and Public mechanisms negatively affect reducing vulnerability as expected poverty on physical and mental health. As shown in column (4) of Table 4, the dy/dx of Government subsidies and Public mechanisms (GS and PM) are − 0.0451 and − 0.0015, respectively (*ρ* < 0.01, *ρ* > 0.1). Among them, Government subsidies had a significant negative correlation with vulnerability as expected poverty on physical and mental health. The latter indicates the regression results can fully support that the level of government subsidies has a certain degree of marginal effects on reducing vulnerability as expected poverty on physical and mental health. Moreover, there is a non-significant negative correlation between public mechanisms and vulnerability as expected poverty on physical and mental health, indicating that public health provision mechanisms for public health services for urban and rural residents are not effective in mitigating health vulnerability by formulating public policies from the supply perspective only. Thus, there is the need to consider the different levels of demand for public health services from both urban and rural residents. This suggests the importance of including the residents’ demand for health income elasticity in the regression results in Table 5.

**Table 5:** The interaction effect of the joint variable

Variables	GS*E_H	PM*E_H
Wald(Prob.)	0.0016^***^	0.0266^**^
Chi2 (1)	10.01	4.91

*Source*: China Family Panel Studies (CFPS) 2018 [64]

*Note*: *、**、*** represented statistic indicators significantly at 10, 5, and 1%, respectively

### The marginal effects of covariates and area on VEP-PH&MH

The dy/dx in Table 4 (items 3–14) show that marriage and age have a significant negative association with VEP-PH&MH (dy/dx = -0.0110, − 0.0010, *ρ* < 0.1, 0.01, dy/dx = the probability of marginal effects), and family size have a significant positive association with VEP-PH&MH (dy/dx = 0.0222, *ρ* < 0.01). While gender and education, identification presented non-significant in the first stage of logistic regression. These results suggest differences in government subsidies and public mechanisms for reducing health vulnerability by personal information identification, age, family size, marriage. Social status had a positive effect on mitigating VEP-PH&MH (dy/dx = − 0.0004, *ρ* < 0.05). Thus, an effective reduction of health vulnerability through social relationship identification should be based on good social status and family economic relationships. Good lifestyle habits also had a significant positive effect on reducing VEP-PH&MH (dy/dx = 0.0028, 0.0173, − 0.0003, *ρ* > 0.1, *ρ* < 0.05, *ρ* > 0.1), including not smoking, drinking less alcohol, and exercising regularly. Therefore, people should be encouraged to adopt good lifestyle habits. The higher the patient’s credit to the doctor’s medical institution, the more beneficial it is to reduce VEP-PH&MH (dy/dx = − 0.0007, *ρ* > 0.1), indicating the importance of improving the medical environment and treatment to enhance the patient’s trust in the doctor and medical institution. Yet, the identification of patients' trust in physicians showed a less than significant effect, which may be attributed to the size of the sample selected. Individual residents in urban areas had a significant negative association with VEP-PH&MH (dy/dx = − 0.0167, *ρ* < 0.01), suggesting that compared with urban areas, residents in rural areas are more likely to suffer from VEP-PH&MH, showing the non-equity between urban and rural areas in health vulnerability.

### Second-layer multivariate logistic model regression analysis: marginal effects of government subsidies and public mechanisms on vulnerability as expected poverty on physical and mental health (VEP-PH&M) after adding income elasticity of physical and mental health demand

The following aspects will be analyzed in this section (i) the marginal effects of government subsidies and public mechanisms, and (ii) the marginal effects of covariates and area, both related to VEP-PH&MH after adding income elasticity of physical and mental health demand.

### The marginal effects of government subsidies and public mechanisms on VEP-PH&M (after adding income elasticity of physical and mental health demand)

Before analyzing the marginal effect of the interaction effects between the two interactive variables of government subsidies, public mechanisms with the income elasticity of demand for health care, GS*E_H and PM*E_H, respectively, in reducing the vulnerability as expected poverty on physical and mental health, it is necessary to line-check whether the variables composed of interactive items have an interaction effect. Thus, to test whether the interactions are significantly combined, by testing the original hypothesis that the coefficient of the interaction variable is all 0. As can be seen in Table 5, the results shows that the Prob. value of the GS*E_H and PM*E_H is less than chi2, and both are significant at the confidence level of 0.01 and 0.1, respectively. Hence, rejecting the assumption that the coefficient of the interaction term is all 0. It is indicated that the joint significance test of this interaction item has passed.

Table 6 reports the multivariate logistic regression model obtained after adding the income elasticity of demand for health for both urban and rural residents. First, we mainly observe whether the relationship between government subsidies and public mechanisms, the two key explanatory variables in the present work, and VEP-PH&MH changes significantly after considering the different levels of demand for health among urban and rural residents. Then, from the regression results shown in items 3 to 5 of the fourth column in Table 6, the income elasticity of demand for health care of residents has a significant negative effect on reducing VEP-PH&MH, i.e., the increased degree of demand for health by individual resident is more likely to reduce the health poverty vulnerability of the residents (dy/dx = − 0.3527, *ρ* < 0.01). Therefore, as residents’ demand for health goes up, the marginal effects of government subsidies and public mechanism instruments on improving residents’ health poverty vulnerability strengthen. Table 6 shows that given the individual effects of government subsidies and public mechanisms, after the inclusion of the income elasticity of demand for health care, the implementation of both government subsidies and public provision policy have marginal effects on reducing VEP-PH&MH of residents. Additionally, the probability of the effect of reducing VEP-PH&MH increases by 3.3% and 0.46%, respectively, in comparison with the first-layer regression result. On the other hand, the results indicate that the interaction effects of residents’ income elasticity of demand for health care with government subsidies and public mechanisms were significant and negative, respectively. The latter indicates that the interaction effect between GS and E_H has a significantly positive effect, explaining that the main effect GS, on the contrary, has a significantly less inhibitory effect on VEP-PH&MH with the co-action of the variable E_H, suggesting that there is a difference between the extent of GS policy implementation and the residents' increasing demand for health (dy/dx =0.0744, *ρ* <0.01). In contrast, the interaction effect between PM and E_H has a significantly negative effect, indicating that the main effect PM has a significantly stronger inhibitory effect on VEP-PH&MH with the co-action of the variable E_H, signifying that a balance between the supply policy of public health resources and the degree of residents' demand for health is being continuously followed. And when government authorities apply the same unit of public service provision to urban and rural residents, if the individual household’s degree of health need keeps increasing, public mechanisms intervention can effectively reduce the health vulnerability to poverty in 1.28% (dy/dx = − 0.0128, *ρ* < 0.01). The results suggest that local governments should focus on the different levels of residents’ health needs in mitigating health vulnerability through government subsidies and public mechanism instruments and enhance the targeting of local governments in implementing policies.

**Table 6:** Variable description and statistics

Item	Identification VEP-PH&MH	Variables	dy/dx (%)	z
1	GS	Government subsidies	-0.0781^***^	-3.88
2	PM	Public mechanism level	-0.0061	-1.18
3	HE	E_H	-0.3527^***^	-20.96
4	IVS	GS*E_H	0.0744^***^	3.16
5		PM*E_H	-0.0128^**^	-2.22
6	PII	Gender	-0.0117^***^	-2.67
7		Age	0.0005^**^	2.48
8		Family size	0.0213^***^	15.66
9		Marriage	-0.0189^***^	-3.74
10		Education	-0.0020^***^	-4.21
11	SRI	Job satisfaction	-0.0020^*^	-1.64
12		Social status	-0.0010	-0.56
16	LHI	Smoke	-0.0007	-0.16
17		Drink	0.0050	0.97
18		Exercise	-0.0004^*^	-1.74
19	PT	Patient trust	-0.0004	-0.59
20	UDV	Urban	-0.0101^**^	-2.57
21	Number of Sampling		8831	
22	Province		Yes	
23	Prob >F		0.000	

*Source*: China Family Panel Studies (CFPS) 2018 [64]

*Note*: *、**、*** represented statistic indicators significantly at 10, 5, and 1%, respectively

The regression results for covariates and areas in Table 6 differ from those reported in Table 4 in that the gender variable in personal information identification , and the exercise variable in the identification of lifestyle habits change from insignificant in Table 4 to significant in Table 6. It shows that the inclusion of the E_H leads to a more significant marginal effect in mitigating health vulnerability for those who are highly educated, enjoy exercising, and with male characteristics. In addition, the age identification variable varies from being positively significant in Table 4 to be negatively significant in Table 6. Thus, the significant relationship between the other covariates and area variables and VEP-PH&MH is consistent with the results reported in Table 4, indicating the importance of government subsidies and public mechanisms in reducing health vulnerability after controlling for the covariates and areas variables.

### Comparing the marginal effects of physical and mental health vulnerability between urban and rural residents

Table 7 shows regression results indicating that there was inequity between urban and rural residents for the intervention mechanism to reduce health poverty vulnerability. In relation to the two main variables, government subsidies and public mechanisms, government subsidies were much more likely to reduce VEP-PH&MH in urban residents than in rural residents, and the effect of this intervention mechanism was not significant in rural residents (urban and rural: dy/dx = −0.1198, −0.0077, *ρ* < 0.01, *ρ* > 0.1). The probability of implementing public mechanisms to reduce VEP-PH&MH of rural residents was much higher than that of urban residents, and it is interesting to note that the intervention of public mechanisms showed a positive and insignificant effect on VEP-PH&MH of urban residents in this study (urban and rural: dy/dx = −0.0263, −0.1124, *ρ* > 0.1, *ρ* < 0.01). This may be related to the government’s failure to develop specific policies for public service provision. Therefore, a follow-up attempt was made to take into consideration the degree of health demand of urban and rural residents in this study, i.e., to test whether the income elasticity of demand for health care and its interactive effects with government subsidies and public mechanisms, respectively, also showed urban-rural differences in the inhibiting effect on VEP-PH&MH. It was found that the larger the level of demand for health, the higher the effect of reducing VEP-PH&MH was significant for both urban and rural individual households, and more specifically, the probability of reducing VEP-PH&MH was higher for urban residents than for rural residents (urban and rural: dy/dx = − 0.4178, − 0.3498, *ρ* < 0.01). Then, we consider the effect of interaction effects. It revealed that there has been a significant inequality between urban and rural residents in the intervention of GS and PM to reduce VEP-PH&MH. Considering the disparity in individual health needs between urban and rural residents, an increase in GS under the cross-effect of increasing desire for health needs among urban residents instead leads to a likelihood of an 11.41% increase in VEP-PH&MH among urban residents (dy/dx= 0.1142, *ρ* < 0.01), and this interaction effect is not significant in rural areas. The enhanced PM effect had a 12.76% probability (dy/dx=0.1276, *ρ* <0.01) of reducing VEP-PH&MH among rural residents under the same interaction effect, and this interaction effect was insignificant in urban areas. The former result may be closely related to factors including the degree of individual physical and mental health needs of urban and rural residents and the matching ratio of government subsidies to the health expenditure gap. The probability that government subsidies can reduce VEP-PH&MH of urban residents becomes lower in the face of intense competitive pressures and increasing health risks of physical and mental co-occurrence. Compared to urban areas, rural individuals face less deprivation of physical and mental health than urban individuals even though their health needs are also increasing each year. Therefore, the increase in government subsidies may contribute to the reduction of VEP-PH&MH to some extent by increasing the ability of rural residents to cope with health risks. The latter result may be due to poorer health care provision in rural areas, and the government may increase the supply of health resources to rural areas, but the public mechanisms implemented cannot balance the extent of the population's needs compared to the increasing health needs of rural residents.

**Table 7:** Urban-rural inequality

Item	Identification VEP-PH&MH	Variables	Urban		Rural	
			dy/dx (%)	z	dy/dx (%)	z
1	GS	Government subsidies	-0.1198^***^	-4.63	-0.0077	-0.26
2	PM	Public mechanism level	-0.0263	-0.93	-0.1124^***^	-2.96
3	HE	E_H	-0.4178^***^	-13.22	-0.3498^***^	-8.19
4	IVS	GS*E_H	0.1142^***^	3.73	-0.0035	-0.10
5		PM*E_H	-0.0287	-0.86	0.1276^***^	3.00
6	CV	Covariates	Yes		Yes	
7	Number of Sampling		5036		3795	
8	Province		Yes		Yes	
9	Prob >F		0.000		0.000	

*Source*: CFPS 2018 [64]

*Note*: ^*^、^**^、^***^ represented statistic indicators significantly at 10, 5, and 1%, respectively

### Comparing the marginal effects of physical and mental health vulnerability among eastern, central, and western residents

Table 8 illustrates the output of the regressions, showing the non-equality between regions in respect to the intervention mechanisms to reduce VEP-PH&MH. This work has divided China’s urban and rural areas into eastern, central, and western regions from high to low, based on the regional features of China’s economic zones. A further logistic regression analysis was conducted on the marginal effects of VEP-PH&MH in these three regions. In terms of the two main variables, government subsidies and public mechanisms, the findings showed that the suppressive effects of government subsidies and public mechanisms on resident health vulnerability varied significantly across the three regions. Thus, the probability of the restraining effect of government subsidies on VEP-PH&MH of residents being significantly stronger in the central region than in the eastern region, while the impact of the government subsidies application was not significant in the western region. The public mechanism had a significant marginal effect on reducing resident health poverty vulnerability in the central and western region. In addition, there are regional disparities in the curbing effects of the key variables in this paper, i.e., the income elasticity of demand for health care and its interaction effects with government subsidies and public mechanisms, respectively, on VEP-PH&MH. Firstly, concerning the marginal utility of the individual variable of income elasticity of demand for health care on VEP-PH&MH, the results showed that the greater the degree of individual households’ demand for health, the higher the probability of reducing VEP-PH&MH for residents in the eastern region than in the western regions. Secondly, regarding the marginal effect of the two interaction terms on VEP-PH&MH, it is found that the interference effects of the implementation strength and effects of GS and PM on the suppression of VEP-PH&MH in the eastern, central, and western regions show significant regional inequalities as the health needs of residents continue to increase. The marginal effects of GS on the suppression of VEP-PH&MH of residents in the eastern and central regions under the condition of the increasing health demands was weakened, specifically, the increasing expenditure on physical and mental health needs of residents in the east and central regions under the condition of increasing degree of physical and mental health needs far exceeds the subsidy effect brought by GS, resulting in a significant negative effect of GS on reducing VEP- PH&MH of residents in the east and central regions. In contrast, GS has an effect of reducing VEP-PH&MH for residents in the western region, but the extent of the effect is not significant (east, central, and west: dy /dx= 0.0813, 0.1171, -0.0159, *ρ* < 0.01, *ρ* > 0.1). The high level of economic development and competitive pressures in the eastern and central regions compared to the western regions have led to increasing real and opportunity costs spent on health by their regional residents. Therefore, the effectiveness of GS in suppressing VEP-PH&MH among residents of the eastern and central regions may be substantially reduced. Another association effect PM implementation under the influence of residents' increasing health demand has a diluting marginal effect on suppressing the VEP-PH&MH of residents in the central and western regions. Another association effect PM implementation under the influence of residents' increasing health demand has a diluting marginal effect on suppressing the VEP-PH&MH of residents in the central and western regions. Meanwhile, PM has an effect on reducing VEP-PH&MH for residents in the eastern region, but the impact level is not obvious (east, central, and west: dy /dx= -0.0210, 0.0839, 0.1339, *ρ* > 0.1, *ρ* < 0.01,  *ρ* < 0.01). The supply coverage of health care in the central and western regions, especially in the western region, would be less than that in the eastern region, and when the residents' demand for health resources outweighs the public resource supply, then the residents' exposure to health risks would be raised, and thus, the effect of PM on reducing the residents' VEP-PH&MH could be softened.

**Table 8:** East-med-west

Item	Identification VEP-PH&MH	Variables	East		Med		West	
			dy/dx (%)	z	dy/dx (%)	z	dy/dx (%)	z
1	GS	Government subsidies	-0.08841^***^	-2.81	-0.1255^***^	-3.55	-0.0071	-0.18
2	PM	Public mechanism level	-0.01579	-0.48	-0.0778^**^	-1.98	-0.1111^**^	-2.29
3	HE	E_H	-0.4332^***^	-12.29	-0.3807^***^	-7.86	-0.3758^***^	-6.69
4	IVS	GS*E_H	0.0813^**^	2.18	0.1171^***^	2.95	-0.0159	-0.34
5		PM*E_H	-0.0210	-0.53	0.0839^***^	1.91	0.1339^**^	2.38
6	CV	Covariates	Yes		Yes		Yes	
7	UDV	Urban	Yes		Yes		Yes	
8	Number of Sampling		4259		2508		2064	
9	Province		Yes		Yes		Yes	
10	Prob >F		0.000		0.000		0.000	

*Source*: CFPS 2018 [64]

*Note*: ^*^、^**^、^***^ represented statistic indicators significantly at 10, 5, and 1%, respectively

### Differences in economic levels, according to region and urban-rural economic disparity

**Fig. 2:**
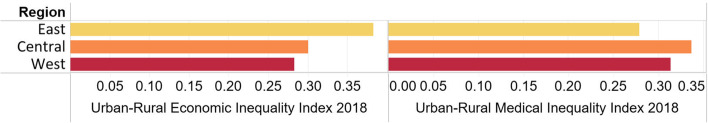
Difference between urban and rural EII and MII in the eastern, central, and western regions of China in 2018 (EII = Economic Inequality Index, MII = Medical Inequality Index). Note: Based on the methods presented by [65, 66], the equivalence is shown by: ${g}_{ME}\left(X,W\right)=-E\left[\log p\left(g(Wx)\right)\right]\triangleq H(z)=E\left[\sum_{i=1}^m\mathit{\log}p\left({y}_i\right)\right]+\mathit{\log}\left|\mathit{\det}W\right|-E\left[\mathit{\log}p(x)\right]\propto Tlog\left|\mathit{\det}W\right|+\sum_{t=1}^T\sum_{i=1}^m\mathit{\log}p\left({w}_i{x}_t\right).$ Where the maximum entropy (ME) based ICA method meets the perspective of a neural network by estimating the demixing matrix *W*_*ME*_, which maximizes the entropy H(·) of the nonlinear outputs *z* = *g*(*y*) of a neural network. In particular, using the cumulative distribution function for a nonlinear function g(·), i.e., ${g}_i^{\prime}\left({y}_i\right)=p\left({y}_i\right)$, assures the equivalence between the ME method using *W*_*ME*_

**Fig. 3:**
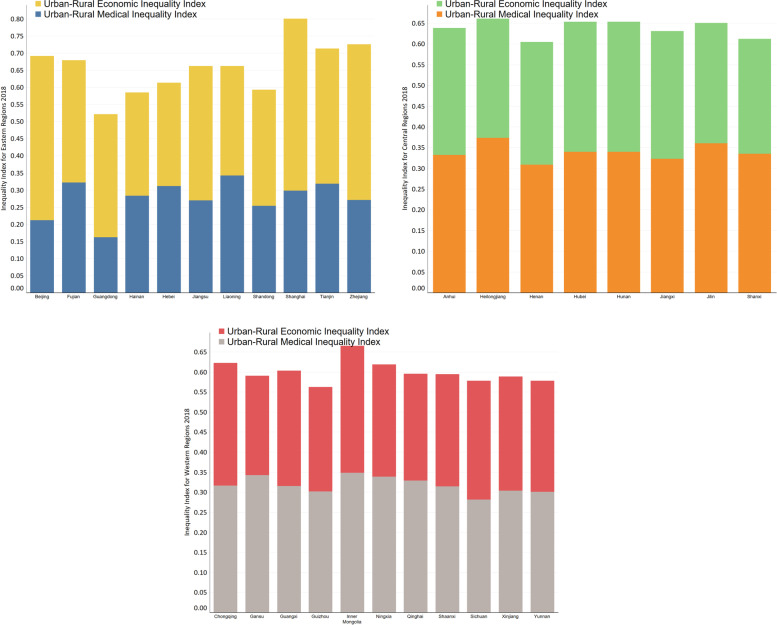
The gap of the urban-rural economic index and public health service index for each province and city in the three regions of China. Note: Based on the availability of data, only thirty provinces of China are shown in the figures for this study. Based on the methods presented by [65, 66], the equivalence is shown by: ${g}_{ME}\left(X,W\right)=-E\left[\mathit{\log}p\left(g(Wx)\right)\right]\triangleq H(z)=E\left[\sum_{i=1}^m\log p\left({y}_i\right)\right]+\mathit{\log}\left|\mathit{\det}W\right|-E\left[\log p(x)\right]\propto Tlog\left|\mathit{\det}W\right|+\sum_{t=1}^T\sum_{i=1}^m\mathit{\log}p\left({w}_i{x}_t\right).$ Where the maximum entropy (ME) based ICA method meets the perspective of a neural network by estimating the demixing matrix *W*_*ME*_, which maximizes the entropy H(·) of the nonlinear outputs *z* = *g*(*y*) of a neural network. In particular, using the cumulative distribution function for a nonlinear function g(·), i.e., ${g}_i^{\prime}\left({y}_i\right)=p\left({y}_i\right)$, assures the equivalence between the ME method using *W*_*ME*_

We also found that differences in economic levels across regions also acted on the implementation effects of government subsidies and public mechanisms on reducing VEP-PH&MH among urban and rural residents. Under the regression results based on the second-level logistic model, after controlling for covariates as CV and UDV, in a condition where families have an increasing need for physical and mental health, the study found that government subsidies exerted a significantly lower inhibitory effect on VEP-PH&MH in the eastern and central urban areas than in the western areas. The implementation effect of public mechanisms in the central and western rural areas significantly weaker contributes to reducing VEP-PH&MH among its residents. In contrast, the implementation effect in the eastern regions is insignificant. This may be caused by the increased economic and public service disparity between different regions in China. We applied the coefficient of variation and information entropy method to divide China’s economic belt into eastern, central, and western regions from high to low. Thus, measuring economic inequality indicators of China’s urban-rural and public health service supply indicators based on the personal distributable income of urban and rural areas, respectively [65, 66] (Figs. 2 and 3). Figure 2 compares urban-rural economic disparity and public health service disparity among China’s eastern, central, and western regions in 2018. Figure 3 shows the urban-rural economic index and public health service index gap for each province and city in China’s eastern, central and western regions. Thus, the urban-rural economic disparity in the eastern and central region are more significant than in the western regions. The increased urban-rural economic disparity in the eastern region has caused the rural residents to strengthen their demand for government transfer payments. Besides, urban residents' pursuing physical and mental health may also be greatly exceeding their consumption ability for health demands, causing the effect of government subsidies in the east and central area to fail to cover the increasing level of health needs of urban residents in the region. In contrast, the urban-rural economic disparity in the western region is relatively insignificant. Consequently, the rural residents reflect to a lesser extent the suppressive effect of government transfer payments acting on VEP-PH&MH, which explains the eastern urban and rural residents having a higher risk capacity to reduce health vulnerability after receiving government subsidies than in the central and western regions. The urban-rural public health service gap in the central and western region are more significant than that in the eastern regions. The widening of the urban-rural public health service gap in the central and western region illustrate the severe lack of public health service investment in local governments in the central and western rural areas, which explains that local government input in public health care services in rural areas of both central and western regions are severely inadequate on one hand, and residents in central and western regions suffer from lower awareness of health vulnerability risks than those in eastern regions on the other hand. It suggests that we should focus on the discrepancy of supply and demand for physical and mental health among eastern, central, and western regions, and reinforce the precision of government subsidies and public mechanisms to intervene in VEP-PH&MH.

## Discussion

In this study, the multidimensional poverty and vulnerability of physical and mental health of Chinese urban and rural residents were estimated, using CFPS data from a cross-sectional survey in 2018. Moreover, the marginal effects and magnitude of the impact of GS and PM on reducing VEP-PH&MH in China urban and rural areas before and after the inclusion of income elasticity of health demand, through a multidimensional logistic two-tier model were explored. There were three critical findings from our study.

### First level logistic regression

The results of the first level logistic regression study showed that the implementation of government subsidies and public mechanisms in rural and urban areas provided significant support for reducing the marginal effect of VEP-PH&MH, and these results are consistent with previous studies [19, 36, 67]. After controlling for PII, SRI, LHI, and PT variables that affect VEP-PH&MH, we noted that GS can directly affect VEP-PH&MH, and the results showed that an increase in government subsidies have a significant positive effect on reducing physical and mental health vulnerability of individual residents in both urban and rural areas. Consistent with existing studies, the findings of this work support the hypothesis that as government subsidies increase and government public transfers increase, the income level and coping capacity for health risks of urban and rural residents who suffer from health vulnerability exploitation increase relatively, and the ability to spend on future physical and mental health vulnerability increases accordingly, and VEP-PH&MH of individual residents will decrease [19, 21, 68]. Considering the direct effects of government subsidies, mainly in the nature of specialized transfers, implying that the central government decentralizes transfer revenues from health care-specific services directly to local governments for increasing the payments of local residents [69, 70]. Meanwhile, government subsidies have spillover effects. Embodied at the macro level, central public transfers indirectly lead local governments to allocate more financial resources to public services aimed at reducing vulnerability on physical and mental health. At the micro level, specific categories of government subsidies also have spillover effects on the inhibitory effects of VEP-PH&MH of beneficiary groups, for instance, government delegated pension subsidy for the elderly, and social security subsidy for sub-healthy working groups to enhance physical and mental of health security. The study indicates that public mechanisms have some effect but not significant impact on reducing VEP-PH&MH, which on the one hand shows that the policy orientation and goal of public mechanisms enhancement on reducing physical and mental health vulnerability of individual residents in urban and rural areas is correct, on the other hand, it also empirically reflects that the implementation of public mechanism must be based in considering the different levels of health demands of individual residents, this is consistent with the findings of other researchers [68]. As urbanization in China advances at a rapid pace, the individual demands of urban and rural residents for local health service supply, including the demand for access to local public hospitals and the provision of local health resources, have gradually strengthened and emerged in a varied and diversified trend.

### Second level logistic regression

The second level logistic regression study results showed that the income elasticity of demand for health care and the interactive effects of the government subsidies with the income elasticity of demand for health care and public mechanisms with the income elasticity of demand for health care are key factors in effectively reducing VEP-PH&MH. We found that, based on the conclusion obtained from the first level of logistic regression, the risk of VEP-PH&MH is closely related to the income elasticity of demand for the health of urban and rural residents. The latter indicates that residents gradually strengthen their awareness of their health needs, prompting them to rationalize the ratio of personal income to health expenditure, by consciously increasing their investment in their physical and mental health, then the vulnerability to physical and mental health and the risk also has a positive inhibitory effect [40]. We also found that the interactive impact of the government subsidies and public mechanisms with the income elasticity of demand for health care can affect VEP-PH&MH through the implementation of local government subsidies and public mechanisms by taking into account the different levels of residents’ health demands. The effect of government subsidies and public mechanisms on VEP-PH&MH involves the interaction of the mediating variable, the income elasticity of demand for health care with it, so that local governments, after taking into account the different levels of residents’ health needs. By enhancing the targeting of government subsidies and public mechanisms formulation and implementation, resulting in the implementation of two public policies, government subsidies and public health service provision, under the influence of the mediating effect of the income elasticity of demand for health care, would effectively improve the vulnerability of Chinese urban and rural residents to physical and mental health [20, 71]. Interestingly, after controlling for regional covariates that identify urban and rural areas (IIR), we find that urban residents are more proactive and motivated to reduce the risk of VEP-PH&MH than rural residents, yet the demand-spending capacity imbalance may lead to a weaker effect of curbing health vulnerability among rural residents. This may be related to inter-economic inequalities between urban and rural areas and regional inequalities in public health service provision, which is consistent with the findings of other researchers [19, 72].

### Comparison of the different effects on improving VEP-PH&MH

Based on the second level of the logistic regression model, we empirically analyzed and compared the extent of the effects of the income elasticity of demand for health care, and the interactive variables of the government subsidies with the income elasticity of demand for health care and public mechanisms with the income elasticity of demand for health care on improving VEP-PH&MH in different regional contexts in urban and rural areas. It was found that the weakening inhibitory effect of government subsidies on VEP-PH&MH was much higher for urban residents than for rural residents. In contrast, the positive effect of public mechanisms implementation on reducing VEP-PH&MH was more significant for rural residents than for urban residents, probably because the high competitive pressure in cities and urban work and living environment stress. Moreover, the probability of urban residents suffering from the risk of being sub-healthy and having chronic diseases has been increasing yearly, causing the demand level for physical and mental health to rise yearly. Thus, government subsidies have less effect in reducing the health vulnerability of urban residents [73]. At the same time, the issue of imbalance is a major cause of the urban-rural gap in developing countries. The contemporary pattern of economic development often results in a “core-marginal” outcome, where economic growth in the center comes at the expense of benefits in the periphery. The faster the center develops, the more the periphery is suppressed, so that residents in the periphery are more vulnerable to poverty [40, 72]. Due to China’s unique urban-rural dualistic structure, local governments tend to invest the dividends of public mechanisms and public services in urban areas to enhance GDP competitiveness, resulting in greater inequality of public services between urban and rural areas and insufficient investment in public services in rural areas, which is consistent with the findings of other studies [73, 74]. This also explains the effect that has increased the implementation of public governmental mechanisms in rural areas, i.e., increasing their public health service investment, has a significant dampening effect on the health vulnerability of rural residents.

### Considerations on the health poverty vulnerability in developing countries in the current global context

The results of the study can provide some insights for developing countries to improve the health poverty vulnerability of the population in the current worldwide context. At first, the current state of health poverty vulnerability deprivation of urban and rural residents confirms the vulnerability of these residents to forgo physical and mental health treatment and is likely to fall back into poverty. This is aggravated by the current events linked to the worldwide scenery of outbreaks, a recent pandemic, regional wars and climate change [75, 76]. The COVID-19 pandemic generated over 5 million deceased, mostly in vulnerable regions in developing countries such as in Africa [16, 66]. An important share of the deceased were individuals who contributed to their families’ income but didn’t get health care due to high costs [15]. A way to attenuate the impact and consequences of these events on poverty consists of the local government public sector facilitating basic health care needs. The latter can be applied by giving direct government subsidies and developing public service provision mechanisms, such as issuing free health care vouchers for their use and peer-to-peer access to residential areas for health care services. This approach is used in many countries to reduce health disparities between different income groups [77, 78]. Nevertheless, the accelerated pace of these unexpected events worldwide, aggravated by climate change, indicate that these types of approaches could be insufficient [14, 79].

The second level of logistic model regression indicates that whether controlling for urban and rural areas or by dividing the eastern, central, and western regions, local governments (in the case of China), can more effectively identify the different degrees of individual residents health needs. This can be performed between diverse urban and rural areas and regions after strengthening the target of public policies. Moreover, this could be used as a mediator to act on government subsidies and public mechanisms, and target different groups of people to develop and implement targeted public policies, specifically for the groups in risk of physical and mental health vulnerability. The latter shows some unpredictability in the current worldwide context. Hence, rural residents, poor and vulnerable residents, e.g., difficult to adopt targeted public policy groups in less developed areas, should be the primary focus of attention [19, 68]. This is linked to the weakening effect of inequality in distributable income, and inequality in public service supply between urban and rural areas, and between regions on the physical and mental health vulnerability of residents. Thus, paying attention to the goal of income and public service equalization, and guarding against the vicious circle of physical and mental health vulnerability and return to poverty [40].

Mental health is linked in several cases to work proficiency and the possibility of health poverty vulnerability [41]. Finally, research results should continue to be accumulated on the crucial value of poverty vulnerability in public services. At present, there is little research on the current situation and influencing factors of poverty vulnerability in public services such as health care in developing countries (even more with the current unpredictability due to the events worldwide). Moreover, a comprehensive study of the variables affecting the suppression of poverty vulnerability in the public service sector should be analyzed. Thus, drawing effectively on the domestic and international literature on the value of the health care service sector and considering the current situation of poverty in the public service sector that exists in developing countries themselves [80].

As a final note, some limitations of this study should be acknowledged. First, the data for this study are cross-sectional data for the year 2018 of the study. In this way, the conclusions can only explain the correlation between VEP-PH&MHE and the variables affecting it, while the dynamic trend of the path of influence cannot be determined. Additionally, the estimation and impact factor analysis of the VEP-PH&MHE was affected by the questionnaire structure, data collection availability, and the respondents’ subjective response bias. However, these limitations did not invalidate our work, and the nature of the large sample reduced estimation bias to some extent, as did the use of panel data.

## Conclusion

The advancement in China’s new era of poverty eradication has established the need for a long-term stable mechanism to protect residents from vulnerability as expected poverty on physical and mental health (VEP-PH&MH) from returning to poverty. Public services such as health care are both an important strategy and a challenge for the Chinese authorities and the public sector to consolidate poverty eradication effectively. Performing an isomorphic study of the incidence and intensity of VEP-PH&MH, and the subsequent identification of the main variables associated with VEP-PH&MH, allowed obtaining empirical findings from the study. Mainly, the results of the one-stage regression indicate that government subsidies (GS) and public mechanisms (PM) demonstrate their inhibitory effect on VEP-PH&MH through the control effect of personal information identification (PII), social relationship identification (SRI), health-related lifestyle habit identification (LHI), and point optimal test PT covariates, but only from the government sector. However, it is difficult to conclude whether the marginal utility of GS and PM in reducing VEP-PH&MH is necessarily significant only from the supply perspective of government departments in formulating and implementing public policies. New findings from the two-stage regression results suggest a mediating effect of the income elasticity of residents’ demand for health on the significant impact of GS and PM. The empirical evidence shows that the cross effect of the interaction between government subsidies with the elasticity (GS*E_H), and the interaction between public mechanisms with the elasticity (PM*E_H) is analyzed by scientifically measuring the income elasticity of health demand of urban and rural residents. This analysis is even more difficult in the current worldwide context inserted into the extended COVID-19 pandemic, viral outbreaks, regional conflicts and climate change, thus it should be observed in a multivariable way. As a limited conclusion (due to the current unpredictability), the government public sector should pay attention to the variability of the degree of residents’ demand for health due to the economic and public service differences between urban and rural areas and between different regions. This would help to improve the targeting of the public sector to implement and develop public health mechanisms, so that GS and PM exert their significant inhibitory effects of VEP-PH&MH.

## Data Availability

The data source of this study was a publicly available database, the China Family Panel Studies (CFPS), which was hosted by the China Social Science Survey Center (ISSS) of Peking University.
